# Health Functions of Egg Protein

**DOI:** 10.3390/foods11152309

**Published:** 2022-08-02

**Authors:** Ryosuke Matsuoka, Michihiro Sugano

**Affiliations:** 1 R&D Division, Kewpie Corporation, Tokyo 182-0002, Japan; 2 Kyushu University, Fukuoka 819-0395, Japan; suganomi@deluxe.ocn.ne.jp; 3 Prefectural University of Kumamoto, Kumamoto 862-8502, Japan; 4 Chair of the Japan Egg Science Society, Tokyo 182-0002, Japan

**Keywords:** egg, protein, egg protein hydrolysate, egg white, cholesterol-lowering, visceral fat-lowering, antifatigue

## Abstract

Egg protein is a remarkably abundant source of protein, with an amino acid score of 100 and the highest net protein utilization rate. However, there have been relatively fewer studies investigating the health benefits of egg protein. In this review, we have summarized the available information regarding the health benefits of egg proteins based on human studies. In particular, studies conducted on the characteristics of egg whites, as they are high in pure protein, have reported their various health functions, such as increases in muscle mass and strength enhancement, lowering of cholesterol, and visceral fat reduction. Moreover, to facilitate and encourage the use of egg white protein in future, we also discuss its health functions. These benefits were determined by developing an egg white hydrolysate and lactic-fermented egg whites, with the latter treatment simultaneously improving the egg flavor. The health benefits of the protein hydrolysates from the egg yolk (bone growth effect) and eggshell membrane (knee join pain-lowering effect) have been limited in animal studies. Therefore, the consumption of egg protein may contribute to the prevention of physical frailty and metabolic syndromes.

## 1. Introduction

Chicken eggs are a highly nutritious food, which contain most of the essential nutrients for human life except for vitamin C and dietary fiber [1]. However, as they also contain cholesterol, patients with dyslipidemia may be advised to avoid their consumption. In healthy individuals, eggs have been reported to have no effect on blood lipid content [2]. This is because even if cholesterol is ingested from the diet, the body has mechanisms by which to control its levels by reducing cholesterol synthesis in the liver [3].

The various health benefits of eggs have been demonstrated in the existing literature, such as the improvement of blood lipids due to the intake of the phospholipids found in the egg yolk, the improvement of cognitive function due to the choline in the egg yolk [4,5], and increased bone density due to the calcium derived from the eggshells [6]. In addition, egg whites are characterized by their high dry weight protein content. Previous studies have described the nutritional benefits of protein using whey and soy proteins. In contrast, egg-derived protein research has generally been focused on allergens. However, in recent years, there have been an increasing number of reports describing the nutritional benefits of egg protein.

The amino acid compositions of egg white protein (EWP; dried egg white), casein, and soy protein isolate are shown in Table 1 [7]. One characteristic of EWP is that it contains approximately the same amount of branched-chain amino acids as milk protein. It also has more sulfur-containing amino acids than other protein sources. The amino acid score for EWP is 100, and its net protein utilization is reported to be higher than that of whey protein [8]. Due to the high quality of EWP, in animal experiments, the consumption of EWP was found to increase body protein [9]. Furthermore, body fat was found to decrease, indicating that EWP is expected to affect health by positively impacting metabolic syndrome. Proteins are also present in eggshell membranes, and these have been the subject of various functional studies [10,11]. In this review, we will introduce the health benefits of egg proteins from a nutritional perspective, with a focus on the existing evidence for EWP, which has been reported in humans.

**Table 1:** Amino acid compositions of casein, dried egg white, and soy protein isolates (g/100 g) [7].

	Casein	Dried Egg White	Soy Protein Isolate
Ile	5.0	4.4	4.0
Leu	8.5	7.3	7.0
Lys	7.2	6.1	5.5
Met	2.7	3.2	1.1
Cys	0.4	2.5	1.1
Phe	4.6	5.1	4.6
Tyr	5.2	3.9	3.5
Thr	4.0	4.0	3.7
Trp	1.1	1.3	1.2
Val	6.2	5.8	4.1
His	2,7	2.1	2.4
Arg	3.4	5.0	6.9
Ala	2.7	5.3	3.6
Asp	6.3	9.3	10.0
Gul	19.0	12.0	17.0
Gly	1.7	3.2	3.6
Pro	10.0	3.3	4.7
Ser	5.2	6.0	5.1

Basic research, such as animal studies and component analysis, has been the primary method of research to determine the health benefits of egg proteins. The health benefits of egg protein hydrolysate and ovotransferrin have been reviewed [12,13,14,15], although based on the results of basic research. In one review considering human studies, the health benefits of the egg itself have been reported [16,17]. Alternatively, a review summarizing the efficacy of egg protein in muscle strengthening, the prevention of sarcopenia, and the prevention of metabolic syndrome of EWR is also available [18,19]. To harness the health value of eggs at the societal level, it is important to highlight its effectiveness in human studies, identify the mechanisms of the active ingredients, and to make them easily accessible for consumption as foods and pharmaceuticals. However, review articles covering these contents have not been reported.

**Figure 1:**
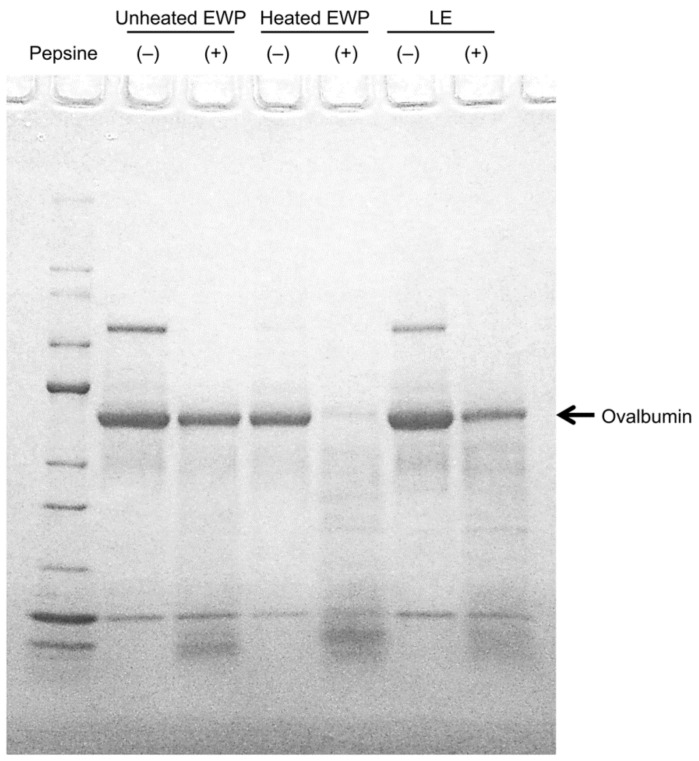
Electrophoresis of unheated EWP, heated EWP, LE, and its pepsin hydrolysates. Note: (+) protein pepsin hydrolysate; (−) protein before acting with pepsin [21].

In addition, it is not comfortable to consume EWP as is, especially egg whites and dried egg whites, due to their physical properties and flavor. Consequently, lactic-fermented egg whites (LE) were developed, which can be consumed without additives by improving the flavor through the lactic acid fermentation of egg whites [20]. LE have almost the same electrophoretic band positions and pepsin degradability as unheated egg whites (Figure 1) [21], making it possible to verify the effects of the EWP in humans using LE. In this review, we summarize the health benefits of egg protein and report on its effectiveness in humans and its mechanisms.

## 2. Muscle Mass and Strength-Enhancing Effects of Egg White Protein in Combination with Exercise

EWP reportedly has protein digestibility corrected amino acid scores (PDCAAS) and digestible indispensable amino acid scores (DIAAS) that are comparable to those of whey protein [22]. In addition, the net protein utilization rate for unheated, half-boiled, and heated states of EWP is reportedly higher than that of whey protein, which is known as a good protein source [8]. In animal studies, the body protein level and gastrocnemius muscle weight in rats fed EWP were found to be significantly increased when compared to those fed casein [9], making it a promising protein supplement for muscle maintenance and building.

Suzuki et al. reported that in addition to mild resistance exercise, 15 g of EWP and 18 g of sugar per day for five weeks increased the isometric muscle strength in the forearm flexors and legs when compared to pre-consumption [23].

When women over 50 years of age were given lactic-fermented egg white (8 g per day as EWP) for eight weeks in addition to twice-weekly fitness club exercise, it was observed that the skeletal muscle weight of the limbs increased significantly in women over 55 years of age when the lactic-fermented egg white intake group were compared to the control group. Furthermore, in these women, the isometric knee extension muscle strength was increased when compared to that before ingestion [24]. This indicates that the ingestion of EWP during exercise increases both the muscle mass and strength.

There are reports, however, that the muscle-mass- and muscle-strength-enhancing effects are stronger for whole egg protein than for EWP, and future studies are expected to resolve this [25,26].

In studies using egg white as a protein supplement, it is not appropriate to use liquid egg white itself due to its unfavorable taste and flavor. Therefore, dried egg white is likely to be used. However, there are numerous issues regarding the flavor and physical properties of dried egg whites when consumed without additives. Consequently, the development of dried egg whites that are easy to consume or the use of LE is desirable.

## 3. Egg White Protein Improves Lipid Metabolism

### 3.1. Visceral Fat Reduction Effects

EWP is classified as a high-quality protein source, and when fed to rats their whole-body protein content was found to be higher when compared to those fed casein. The EWP intake was, thus, shown to increase body protein and decrease total body fat levels [9]. In contrast, when fed the EWP, the triglyceride and leptin concentrations in the rat tail vein plasma were found to be significantly lower in comparison with those fed casein, indicating that EWP could reduce visceral fat levels [9]. Consequently, the effects of EWP on the visceral fat in rats was further investigated and compared with casein. The results indicated that the EWP could significantly reduce white adipose tissue weight and white adipocyte area levels. EWP consumption was also found to result in significantly higher gastrocnemius muscle weight in comparison with casein [9]. This indicates that egg white is a good source of protein that can be utilized to increase body protein, i.e., muscle mass. Since muscle tissue is the primary site of fat oxidation [27], it was hypothesized that the consumption of egg whites may reduce both body fat and visceral fat levels.

**Figure 2:**
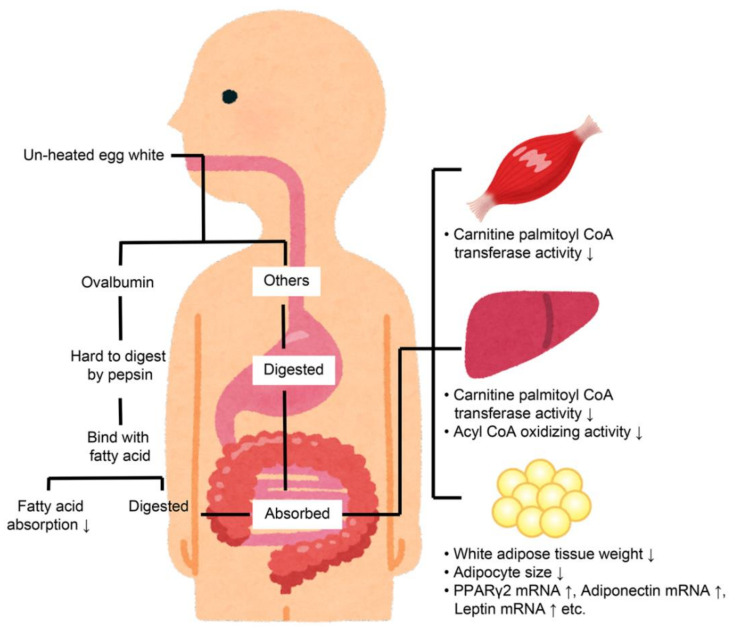
Mechanisms for the visceral fat-lowering effects of egg white protein [9,21,28,29,30].

Unheated egg whites have been used in previous studies, and it was reported that the net protein utilization rates of EWP are comparable between unheated and heated egg whites [7]. Consequently, it was hypothesized that heated egg whites would reduce visceral fat levels in a similar manner to unheated egg whites. The effects of heated egg whites (boiled egg: 95 °C for 10 min) on visceral fat were, thus, compared to unheated egg whites and LE, with casein being used as a negative control. Although the unheated egg whites and LE reduced the visceral fat levels when compared to casein, the heated egg whites did not [20]. These results indicate that there may be mechanisms other than the provided hypothesis, namely that egg whites reduce visceral fat levels by increasing muscle mass. A summary of the known visceral fat reduction mechanisms of EWP is provided in Figure 2.

An allergy study found that the ovalbumin in EWP is not easily degraded by pepsin in unheated egg whites but is easily degraded in heated egg whites [31]. Therefore, if ovalbumin in LE is less likely to be degraded by pepsin, the digestibility of pepsin may be involved in the visceral fat-reducing mechanism. Therefore, when we evaluated the pepsin degradability of ovalbumin in LE, it was found that the ovalbumin in LE was not easily degraded by pepsin [21].

Pepsin is a gastric digestive enzyme, indicating that the main factor in the visceral fat-reducing effect of EWP may be occurring in the gastrointestinal tract. The triglyceride absorption into the lymph of rats undergoing thoracic lymph duct cannulation surgery was also reduced [28]. It was shown that the suppression of lipid absorption by ovalbumin in unheated egg whites may have reduced the visceral fat level. In an in vitro study by Handa et al. (1999), ovalbumin was reported to bind to fatty acids [32]. This indicated that ovalbumin, which is not easily affected by pepsin, can bind to fatty acids degraded by lipase in the digestive tract, thereby suppressing fatty acid absorption.

However, while the suppression of lipid absorption by EWP is approximately 20% higher than that by casein [28], the visceral fat reduction effect is 30% less than that of casein [9]. Despite the differences in experimental conditions, these tests were conducted by feeding diets containing 20% casein or EWP, and the visceral fat reductions by EWP could not be explained solely by the suppression of lipid absorption. Other mechanisms for reducing visceral fat using EWP include increased β-oxidation in the muscle and liver, as well as a reduction in the visceral adipocyte area [9].

The enhancement of β-oxidation in the liver was reported in a study in which rats were fed diets containing 20% casein or EWP. The results showed that EWP consumption resulted in significantly higher activity levels for carnitine palmitoyltransferase and acyl-CoA oxidase, which are involved in β-oxidation in the liver, when compared to casein [9].

The enhancement of β-oxidation in the muscle was identified in a similar study in which rats were a fed diet containing 20% casein or EWP, and the EWP diet was found to increase gastrocnemius muscle mass and showed higher levels of enzyme activities related to β-oxidation in the muscle when compared to casein [33].

Studies investigating the decrease in visceral fat area in rats have also shown an increase in the expression of a series of genes, including peroxisome proliferator-responsive receptor (PPAR) γ2 and adiponectin in visceral adipocytes, as well as improved insulin sensitivity [29,30]. The reductions in visceral fat by EWPs were also thought to be caused by these effects.

The following two possibilities were considered as explanations for the increased β-oxidation in the muscle and liver and the increased gene expression in the visceral adipocytes. The first was the possibility of a secondary effect for lipid absorption suppression. The second was the possibility of a direct effect of the EWP-derived components on the liver, muscle, and adipose tissue. For the latter, the absorption rate of the unheated EWP was reported to be over 90% [21]. In addition, EWP was digested into peptides in the gastrointestinal tract [34]. Therefore, the absorption of peptides derived from EWP may generate these effects. In the liver, soy-derived peptides reportedly enhance the gene expression of enzymes related to β-oxidation in a mouse model for diabetes [35]. For white fat cells, it has been reported that soy hydrolysate enhances the gene expression of PPARγ2, albeit this has only been found for in vitro results [36]. Based on these findings, we expected to find a peptide with similar effects in the EWP. The identification of the peptide sequence that exerts this effect in EWP will be a subject for future studies.

Reductions in visceral fat using EWP in humans have not yet been investigated. Like EWP, lactoferrin and β-conglycinin from soybeans have also been reported to reduce visceral fat [37,38]. Lactoferrin is reported to reduce visceral fat at 300 mg daily and β-conglycinin at 5 g per day [37,38]. The inhibition of lipid absorption has been reported as a mechanism for visceral fat reduction using β-conglycinin [39], and the inhibition of lipid absorption by ovalbumin, ovotransferrin, and lysozyme has been reported as a visceral fat reduction mechanism for EWP [28,40]. To consume 5 g of these three components in equal measure, 8 g of LE as EWP needs to be consumed.

At the same time, the function of ovotransferrin in EWP was found to be similar to that of lactoferrin. If ovotransferrin has the same physiological activity as lactoferrin, it was hypothesized that consuming 3 g of lactic-fermented egg white per day as protein would reduce the level of visceral fat. Based on this, the minimum effective amount of lactic-fermented egg white was determined to be 3–8 g of protein per day.

Therefore, the effects of LE containing 6 or 8 g of EWP per day or 8 g of whey protein per day was evaluated on the visceral fat levels of 22 Japanese adult males (overall) with a BMI ≥ 24 and waist ≥ 85 cm for eight weeks. The results showed that the consumption of lactic-fermented egg white, which was equivalent to 8 g of EWP per day, reduced the visceral fat area when compared to that before consumption. A further analysis in subjects with a BMI > 25 showed that 8 g per day of lactic-fermented egg white as EWP significantly reduced the visceral fat area and visceral fat/subcutaneous fat ratio when compared to pre-consumption levels or when whey protein was used instead [21]. The results indicate that 8 g of EWP per day may reduce the total visceral fat area.

Moreover, in a double-blind, placebo-controlled study, adult men and women with a visceral fat area >100 cm^2^ consumed lactic-fermented egg white (8 g/day as EWP) or 8 g/day of whey protein for 12 weeks. The results showed that the lactic-fermented egg white group significantly reduced the visceral fat area and visceral fat/subcutaneous fat ratio when compared to the pre-consumption and whey protein groups (Table 2). This suggested that the effective amount of EWP to reduce visceral fat was 8 g per day [41].

**Table 2:** Changes in the visceral fat area and ratio of visceral to subcutaneous fat areas in subjects fed LE or milk whey for 12 weeks [41].

		Time (Weeks)	
		0	12
Visceral Fat area (Δcm^2^)	Milk whey	0 ± 0	1.71 ± 4.00
	LE	0 ± 0	−8.89 ± 2.75 *
Visceral/Subcutaneous (Δ)	Milk Whey	0 ± 0	0.0260 ± 0.0187
	LE	0 ± 0	−0.0876 ± 0.0128 *

Mean ± SE of 18 (control) and 19 (LE), *: *p* < 0.05 vs. milk whey. LE: lactic-fermented egg white.

The effective amount of EWP was determined to be 8 g, according to the amount indicated in a pilot study assessment of the minimal effective dose in a double-blind, placebo-controlled study.

The required protein source for LE per day in only egg whites was confirmed to be 8 g of protein, based on an analysis of its protein content.

### 3.2. Cholesterol-Lowering Effects

Although eggs contain cholesterol, they do not necessarily increase serum cholesterol levels. This is because an individual’s genetic factors and diet must also be considered. It is known that some responders are prone to increased serum cholesterol concentrations when cholesterol is ingested [42,43]. Furthermore, dietary influences such as functional components that reduce cholesterol absorption, such as phytosterols, soy protein, or dietary fiber, should be considered [44,45,46,47,48]. Chicken eggs’ functional components, specifically egg yolk phospholipids, have been reported to suppress cholesterol absorption [49]. As for egg whites, in a study of female college students with borderline or mild hypercholesterolemia, Asato et al. reported that 23 g of EWP per day lowered their serum total cholesterol concentrations [50]. Therefore, the authors investigated the mechanism of serum cholesterol reduction via EWP intake, and a summary of this is presented in Table 3.

**Table 3:** Effects of egg white protein on cholesterol metabolism when compared with casein [28,40].

		Cholesterol Level
In vivo (Rats)	Serum	↓
	Hepatic	↓
	Stomach	N.S.
	Intestinal contents (solid)	↑
	Intestinal contents (micelle)	↓
	Intestinal mucosa 1 (upper)	N.S.
	Intestinal mucosa 2	↓
	Intestinal mucosa 3	N.S.
	Intestinal mucosa 4 (lower)	N.S.
	Lymph	↓
	Fecal	↑
In vitro	Micellar solubility	↓
	Bile acid binding	N.S.
	Phospholipid binding	N.S.
	Transfer to triolein	↓
	Water holding capacity	↑
	Settling volume	↑
	Viscosity	↓

↑: increase; ↓: decrease; N.S.: not significant.

Egg white is rich in cystine, a type of sulfur-containing amino acid that reportedly may lower serum cholesterol levels [51]; however, some reports refute this [52]. The effects of cystine on the cholesterol-lowering effects of EWP were investigated. When rats were fed a high-cholesterol diet containing casein or EWP under conditions with or without cystine, no significant effect from the cystine was observed [40].

Avidin in egg white binds to biotin and suppresses biotin absorption. It has been reported that a deficiency of biotin decreases the serum cholesterol concentration. The serum-cholesterol-lowering effect of EWP was compared with that of casein under conditions with or without biotin, and the biotin was not observed to have an effect [40].

Ovomucin in egg white has been reported to reduce cholesterol absorption [53]. Therefore, we investigated the effects of EWP on lipid absorption. When rats were fed a high-cholesterol diet containing casein or EWP, EWP increased the excretion of neutral sterols and bile acids in the feces [40]. EWP was also found to reduce cholesterol absorption into the lymph of rats undergoing permanent lymph duct cannulation surgery. This indicated that the primary mechanism of the EWP effect was to reduce cholesterol absorption [28].

The cholesterol absorption inhibition function of EWP was investigated. High-cholesterol diets containing casein or EWP were fed under meal-feeding conditions, and cholesterol concentrations in the stomach contents, small intestinal mucosa, and small intestinal contents were measured 2 h after consumption [40]. The cholesterol in the stomach contents was not affected by the EWP. The cholesterol contents in the small intestinal mucosa were lower than those with the casein in the upper part of the small intestine, where cholesterol absorption actively takes place [40]. When the small intestinal contents were separated by ultracentrifugation into the aqueous and solid phases to determine the cholesterol contents, the EWP was found to have decreased the cholesterol content in the aqueous phase and increased it in the solid phase [40]. Since cholesterol absorption requires dissolution in bile acid micelles, the results indicated that EWP might reduce the absorption of dietary cholesterol by affecting the bile acid micelles.

The mechanism of cholesterol absorption inhibition by EWP was evaluated in vitro. Since EWP reaches the small intestine after pepsin digestion in the stomach, experiments were performed using pepsin-digested egg white. The solubility of the cholesterol in the bile acid micelles of the EWP pepsin hydrolysates was lower than that of the casein–pepsin hydrolysates [28]. This effect was maintained in the pepsin–pancreatin hydrolysate, although the effect was slightly weakened [28].

The components in the bile acid micelles that were affected by the EWP pepsin hydrolysate were examined, and the hydrolysate was not bound to the bile acids or phospholipids. In addition, the EWP pepsine hydrolysate was found to have a higher water holding capacity, settling volume, and relative viscosity than the casein–pepsin hydrolysate, suggesting that the physicochemical properties of the EWP pepsine hydrolysate inhibited the solubility of the cholesterol in the bile acid micelles. The release of cholesterol monomers from the bile acid micelles was also found to be suppressed [28].

EWP mainly comprises ovalbumin, ovomucoid, ovotransferrin, and lysozyme [54]. The solubility of cholesterol by pepsin hydrolysate in bile acid micelles was examined, and ovalbumin, ovotransferrin, and lysozyme pepsine hydrolysate were found to be effective in the micellar cholesterol solubility [28]. In addition, reconstituted EWP pepsin–hydrolysate residue, which was reconstituted from these four components, inhibited the solubility of cholesterol in the bile acid micelles as much as the EWP, suggesting that ovalbumin, ovotransferrin, and lysozyme were the components involved [28]. Furthermore, because of the high ovalbumin content among these components, ovalbumin was assumed to be the main component of the cholesterol-lowering effects in the EWP.

Furthermore, a study on hamsters demonstrated that heated EWP does not lower the serum cholesterol concentrations [55]. This suggests that the cholesterol-lowering effect of EWP, like the visceral fat reduction effect, may be due to unheated egg whites or LE. Although ovomucin reportedly inhibits cholesterol absorption [47], EWP contains only a small amount of ovomucin, and its contribution to the serum-cholesterol-lowering effect of EWP seems to be low. At this stage, the synthesis and catabolism of cholesterol in the liver and the effects of EWP in small intestinal absorptive cells have not yet been investigated, but are expected to be a focus of future research.

The effective dose of EWP by which to lower serum cholesterol levels in humans is approximately 23 g per day, but whether a lower amount would be effective is currently unknown. To test this, LE were fed at a rate of 4 g (control group), 6 g, or 8 g per day as EWP to borderline or mildly hypercholesterolemic subjects for eight weeks. The results showed that the consumption of 8 g of EWP per day significantly reduced the serum total cholesterol and LDL-cholesterol concentrations when compared to the control subjects. In addition, there was no significant difference in serum total cholesterol and LDL-cholesterol concentrations when 6 g of lactic-fermented EWP per day was consumed when compared to the control subjects (Table 4) [56]. This suggests that the minimal effective dose of EWP to reduce serum total cholesterol and LDL-cholesterol concentrations is 8 g/day.

**Table 4:** Changes in the serum total cholesterol and LDL-cholesterol levels in subjects fed LE (as 4 g, 6 g, or 8 g of EWP) for 8 weeks [56].

	Egg White Protein	Time (Weeks)		
	Intake/Day	0	4	8
Total Cholesterol (Δmg/dL)	4 g	0 ± 0	−4.46 ± 3.36	3.11 ± 3.38
	6 g	0 ± 0	−7.42 ± 3.76	−5.97 ± 3.53
	8 g	0 ± 0	−11.3 ± 3.63	−11.0 ± 3.74 *
LDL-Cholesterol (Δmg/dL)	4 g	0 ± 0	−7.61 ± 2.98	−2.07 ± 2.91
	6 g	0 ± 0	−8.00 ± 3.51	−9.48 ± 3.12
	8 g	0 ± 0	−10.0 ± 3.00	−13.7 ± 3.74*

Mean ± SE of 28 (4 g), 31 (6 g), and 29 (8 g), *: *p* < 0.05 vs. 4 g.

## 4. Antifatigue Effects of Egg White Protein Hydrolysate

The physiological functions of EWP have been described. However, various health effects of hydrolyzed proteins, which are artificially prepared using enzymes, especially peptides, derived from milk and soybeans have also been reported [57,58,59]. The physiological functions of egg white protein hydrolysate (EWH: mainly Peptifine^®^, Kewpie Corporation, Tokyo, Japan) were, thus, assessed.

The absorption and nutritional value of whey protein hydrolysate with a molecular weight of 500 and EWH with a molecular weight of 2500 were assessed (Egg White Peptide EP-3, Henningsen Foods Inc., Omaha, NE, USA). The results showed that for the EWH, the absorption rate of amino acids into the portal vein was faster than with the whey protein hydrolysate, despite its larger molecular weight. The PDCAAS and DIAAS of egg white hydrolysate as well as whey protein and its hydrolysate. In addition, the net protein utilization was significantly higher for EWH than whey protein hydrolysate [22].

One of the ongoing challenges for EWP is its bitter flavor. To address this, a new hydrolysate that was less bitter was developed by enzymatically degrading egg whites and collecting the soluble fraction (Peptifine^®^) [60].

**Figure 3:**
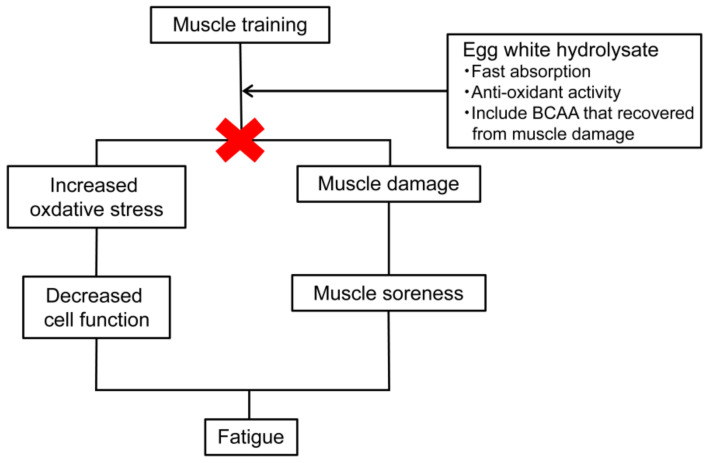
Mechanisms for the antifatigue effects of egg white protein hydrolysate [61,62,63,64,65,70].

Egg whites have high amounts of sulfur-containing amino acids and branched-chain amino acids (BCAA) [61]. Since sulfur-containing amino acids are the source of glutathione [62], they are expected to have antioxidant properties. Antioxidant effects are known to prevent various diseases, including atherosclerotic diseases [63], and have also been reported to lead to antifatigue effects. In addition, BCAAs have been reported to improve muscle fatigue [64], and since the blood BCAA concentration increases when EWP is ingested and decreases after exercise [65], an antifatigue effect of the BCAAs can also be expected (Figure 3).

No antifatigue effects have been reported for proteins, but they have been reported for peptides. Imidazole peptides derived from chickens reportedly have antioxidant properties, and consequently antifatigue effects [66]. Peptides from whey have been reported to increase swimming times in forced swimming mice by chelating radical scavengers and iron [67].

EWH has also been reported to have antioxidant properties [68]. In addition, the pepsin degradation products of egg whites have also been reported to have in vivo antioxidant and antifatigue effects [69].

The effects of EWH (Peptifine^®^) on the swimming times of weight-loaded forced swimming mice were consequently investigated. Seven-week-old male ddY mice were divided into a casein group, an EWH group, and an EWP group and fed diets containing each for 14 days. The swimming times were evaluated daily starting on the 11th day of the study. On the last day of the test, blood samples were taken for a blood test. The results showed that the swimming time of the EWH group was significantly longer than for the casein and EWP groups on the 14th day of the testing. The reason for this was a decrease in the concentration of hexanoyl lysine in the blood of the EWH group. In summary, the egg white hydrolysate was found to prolong the swimming time in forced swimming, and the mechanism of the prolonged swimming time was thought to be due to the antifatigue effect of its antioxidant action (Figure 3) [70].

In healthy marathon runners, 7.5 g of EWH (Peptifine^®^) per day for eight weeks was found to improve the subjective symptoms of physical and overall fatigue when compared to the controls. Additionally, at this time, serum aspartate aminotransferase (AST), alanine aminotransferase (ALT), and lactate dehydrogenase were found to be significantly lower than in the controls at week 8 [71].

Similarly, when marathon runners were given 2.5 g and 5 g of EWH per day for eight weeks, physical fatigue was significantly lower in the group given 5 g of EWH per day when compared to the control group [71].

These results indicate that the minimum effective dose of EWH to exert its antifatigue effect is 5 g. In addition, when athletes were continuously given 5 g of EWH (Peptifine^®^) per day for two weeks, the psychological fatigue was reduced when compared to the control [72]. It was, thus, determined that EWH could be used effectively to improve the exercising capacity of humans, as it is a good source of protein and raw materials for muscles due to its amino acid composition, and it is quickly absorbed and improves physical fatigue through its antioxidant effects.

## 5. Absorption and Physiological Functions of Eggshell Membrane Proteins and Hydrolysate

Eggshells and eggshell membranes are currently unutilized byproducts of chicken eggs that could be potential nutrient resources. For example, eggshells can be used to increase bone density as a calcium-fortifying agent [6]. The eggshell membrane is a thin membrane inside the eggshell, and its main component is protein. Outside the food industry, the eggshell membrane is used as an adsorbent for carbon dioxide and heavy metals [73,74]. Research is ongoing in the food industry to develop a soy sauce using the eggshell membrane [75]. The health effects of the eggshell membrane include anti-inflammatory effects, skin moisturizing effects, and the improvement of knee joint pain [10,11]. The amino acid composition of the eggshell membrane protein is shown in Table 5. The egg shell membrane protein and its hydrolysate include high cystine contents when compared to casein. However, the absorption rate and net protein utilization rate of the eggshell membrane protein (ESM-P) when ingested are unknown. To address this, the absorption of ESM-P and eggshell membrane protein hydrolysate (ESM-H) after digestion were investigated.

**Table 5:** Amino acid compositions of the egg shell membrane protein and its hydrolysate (g/100 g) [76].

	Casein	ESM-P	ESM-H
Ile	4.7	3.2	2.5
Leu	8.4	4.5	3.6
Lys	7.2	3.3	2.4
Met	2.5	3.7	2.9
Cys	0.4	9.7	6.0
Phe	4.6	1.5	1.2
Tyr	5.0	1.7	1.5
Thr	3.8	5.5	4.2
Trp	1.1	3.3	2.4
Val	5.8	6.9	5.3
His	2.7	4.0	2.9
Arg	3.3	7.1	5.4
Ala	2.7	2.6	2.2
Asp	6.4	8.2	6.4
Gul	19.7	12.4	9.5
Gly	1.7	5.8	4.5
Pro	9.5	9.4	7.5
Ser	5.1	5.2	4.0

ESM-P: egg shell membrane protein; ESM-H: egg shell protein hydrolysate.

The absorption rates of the ESM-P and ESM-H were 87.0% and 94.8%, respectively, which was significantly lower than the rate for casein (98.5%). The net protein utilization rates, however, were 84.7% and 84.6%, which were significantly higher than that for casein (75.1%). Although the absorption rate was significantly higher for ESM-H than ESM-P, the net protein utilization rates were not significantly different between the ESM-P and ESM-H [76].

These results indicate that the ESM-P is more than 80% absorbed and utilized by the body. Although there are many unknowns regarding the physiological functions of eggshell membrane peptides, since most of them are taken up by the body, future physiological function studies are expected.

## 6. Health Function of Egg Protein in Basal Research

While human studies on egg proteins, to date, have been limited, further research is expected in the future to address this. Many animal studies have recently been conducted on EWH, and its health effects have been reported. These have included lowering blood pressure [77,78], improving lipid metabolism [79], and improving glucose metabolism [80,81,82], and it is, thus, expected to help prevent and improve metabolic syndrome. In addition, since lowering the serum cholesterol concentration [83], improvements in vascular function and antioxidant and anti-inflammatory actions have also been reported [84,85]; therefore, it is expected to be effectively utilized in the prevention of arteriosclerotic diseases.

EWH has also been reported to improve fatty liver conditions [86]. Furthermore, it is thought to have some positive effects on liver function, as it was found to improve ALT and AST levels when compared to controls in a human study on marathon runners [72]. Furthermore, it has been reported in animal studies to improve neuroprotection and cognitive dysfunction via its antioxidant and anti-inflammatory effects [87,88]. Since it has also been reported to improve mental fatigue in humans, it may also positively affect the cranial nervous system.

Eggshell membrane protein hydrolysate reportedly improves knee joint pain [11], and egg yolk protein hydrolysate reportedly promotes bone growth [89]. Protein hydrolysates are amino acids and peptides. From a nutritional point of view, they also act as raw materials for muscle. In addition, their absorption rate is faster and more efficient because their molecular weight is smaller than those of proteins. Bones, joints, and muscles are key to physical frailty. Especially for older adults with weakened gastrointestinal tract functions, the use of egg protein hydrolysate, which has a smaller molecular weight, could lead to efficient amino acid and peptide supplementation. It has also been reported that long-term EWH consumption can improve physical fatigue [72].

The gap between healthy and average life expectancy rates has widened in recent years. People in developed countries are said to spend, on average, approximately ten years in a bedridden state [90], which places a great burden not only on the person but also on those who provide care. Since the three major factors that shorten a healthy life expectancy are metabolic syndrome, frailty, and dementia, egg protein intake is expected to contribute to extending the healthy life expectancy. EWH has also been reported to enhance immune function [91] and is expected to prevent infectious lung diseases, which are ranked as one of the leading causes of death in many countries.

Recently, it has been reported that egg consumption is an effective way by which to increase protein intake [92]. In addition, it has also been reported that the intestinal environment improves when egg protein and cellulose are consumed together, and a combined effect of egg protein with other foods can also be expected [93].

Furthermore, as mentioned when discussing the visceral fat reduction effect of EWP, we believe that it is also important to find the characteristics of egg proteins with high physiological activity, as the health effects of egg protein change depending on whether they it is heated or cooked [21].

## 7. Discussion

In this review, we have summarized the health benefits of egg proteins and reported its effectiveness and mechanism in humans.

Section 2 summarizes the effects of the consumption of EWP for increasing muscle mass and strength. These are a few of the common benefits of egg protein, as the amino acid balance and its absorption rate seems to be the key factors and have also been reported in other protein sources, such as milk whey and soy proteins [94,95]. However, EWP exhibited a higher net protein utilization rate than milk whey and soy proteins in animal studies [8], suggesting that EWP may be able to increase muscle mass and strength more efficiently compared with milk and soy. Considering the effects of increasing muscle mass and strength, Kato et al. (2010) reported that the effect of EWP alone was not notable, and it seemed to be important to combine it with exercise [23].

Section 3 summarizes the visceral-fat-reducing and serum-cholesterol-lowering effects of EWP. The effects of lactoferrin in milk and β-conglycinin in soybean on visceral fat reduction have been reported [37,38]. Lactoferrin is effective at a dose of 300 mg/day, but it is degraded by gastric acid; thus, it seems to be necessary to take a contrivance to prevent its degradation. Previous reports have also demonstrated the efficacy of lactoferrin in enteric-coated capsules [38]. Soybean β-conglycinin has been shown to reduce visceral fat at a daily dose of 5 g [37]. This amount corresponds to a required daily intake of as much as 25 g of soy protein. EWP reduced visceral fat levels at a dose of 8 g/day [41], suggesting that it could reduce visceral fat levels more efficiently than soy. However, ingenuity may be necessary for its social implementation, since the effect was only evident in unheated egg whites or LE [21]. Additionally, since the visceral-fat-lowering effect of the EWP is based on the inhibition of lipid absorption, it seems likely to lower the visceral fat level more efficiently when consumed in combination with medium-chain fatty acids, catechin, or rose hip tiliroside, which enhance the fatty acid β-oxidation [96,97,98].

Cholesterol-lowering effects have not been reported in milk proteins. The cholesterol-lowering effective dose of soy protein was reported to be 6–10 g/day [99,100], which appeared to be nearly equivalent to that of EWP. Although the impacts of different heating conditions on the cholesterol-lowering effects of EWP have not been investigated, studies in hamsters have reported that heated EWP does not lower serum cholesterol levels [55]. Since the primary mechanism of the visceral-fat-reducing and cholesterol-lowering effects is due to the inhibition of lipid absorption, it was considered that the visceral-fat-reducing and cholesterol-lowering effects of EWP may be present only in unheated egg whites or LE.

The previously reported effects of increasing muscle mass and strength, reducing visceral fat, and lowering cholesterol levels appeared to be equal to or more effective than those of soy and milk proteins, which have been extensively studied. However, both the visceral-fat- and cholesterol-lowering effects of soy and milk proteins have been specifically attributed to specific ingredients that exert effects in smaller quantities than egg white proteins. It is, however, likely that egg white protein at least has these three primary characteristics that seem to boost its nutritional value.

Section 4 mainly summarizes the antifatigue effects of EWH [71]. The effect was attributed to its antioxidant capacity and branched-chain amino acid content. Since the effect was less than that of imidazole dipeptide [65], it is necessary to identify the potent peptide contents of the EWH. However, considering its amino acid score of 100 [22], the effects, such as muscle hypertrophy, seemed to be expected as a good protein source unlike the dipeptide. Finally, Section 6 also describes new functional studies on the plausible multiple effects of EWH. Regardless, further research based on human studies is warranted.

As described in Section 5, ESM-H, which is reportedly absorbed into the body in the first stage [76], also reportedly has moisturizing effects on the skin and helps relieve knee joint pain [10,11]. Although the information on the health benefits of yolk hydrolysate is limited at present [89], future studies focusing on the entire egg protein rather than just the EWP are warranted.

## 8. Conclusions

This review summarizes the health effects of egg proteins, especially EWP, as reported in human studies. Two major functions have been clearly identified: (1) they improve muscle mass and have muscle-strengthening and antifatigue effects when consumed during exercise; (2) they can improve lipid metabolism by reducing visceral fat and lowering serum cholesterol levels. The intake of egg protein may, thus, contribute to the prevention of physical frailty and metabolic syndrome.

## Data Availability

The data presented in this study are available on request from the corresponding author.

## Abbreviations

ALT	alanine aminotransferase
AST	aspartate aminotransferase
BCAA	branched-chain amino acids
DIAAS	digestible indispensable amino acid scores
ESM-P	egg shell membrane protein
ESM-H	egg shell membrane protein hydrolysate
EWH	egg white protein hydrolysates
EWP	egg white protein
LE	lactic-fermented egg whites
PDCAAS	protein digestibility corrected amino acid scores
PPAR	peroxisome proliferator-responsive receptor
